# Comparative Study of Laparoscopic Adnexal Surgery Versus Open Adnexal Surgery for Adnexal Masses

**DOI:** 10.7759/cureus.105768

**Published:** 2026-03-24

**Authors:** Javvaji Kavitha, Shobha Shiragur, Rajasri G Yaliwal, Neelamma Patil, Aruna Biradar, Syeda Atufiyat Amreen

**Affiliations:** 1 Obstetrics and Gynaecology, Shri B.M. Patil Medical College, Hospital and Research Centre, Bijapur Lingayat Development Education (Deemed to be University), Vijayapura, IND

**Keywords:** adnexal mass, gynecological surgery, laparoscopic adnexal surgery, laparoscopy, laparotomy, minimally invasive surgery, open adnexal surgery

## Abstract

Introduction

Adnexal masses are among the most frequently encountered gynecological conditions and may require surgical intervention when symptomatic or suspicious for malignancy. With advances in minimally invasive techniques, laparoscopy has emerged as an alternative to conventional laparotomy, offering potential benefits in terms of perioperative morbidity and recovery. The study aimed to compare laparoscopic adnexal surgery and open adnexal surgery for adnexal masses with respect to intraoperative and postoperative outcomes.

Materials and methods

This randomized controlled trial was conducted in the Department of Obstetrics and Gynecology, Shri B. M. Patil Medical College, Vijayapura, Karnataka, India, from March 2024 to October 2025. Sixty women with adnexal masses requiring surgical management were randomly allocated into two groups: laparoscopic surgery (n=30) and open surgery (n=30). Outcomes assessed included postoperative complications (primary outcome) and secondary outcomes such as duration of surgery and anesthesia, intraoperative blood loss, postoperative hemoglobin levels, analgesic requirement, need for blood transfusion, and duration of hospital stay. Data were analyzed using appropriate statistical tests, and a p-value <0.05 was considered statistically significant.

Results

Both groups were comparable with respect to baseline demographic characteristics. Laparoscopic surgery was associated with significantly lower intraoperative blood loss (p<0.001), reduced postoperative analgesic requirement (p<0.001), shorter hospital stay (p<0.001), and better preservation of postoperative hemoglobin levels (p=0.021). None of the patients in the laparoscopic group required blood transfusion, whereas five (16.7%) patients in the open surgery group did (p=0.018). Although laparoscopic procedures required a longer duration of surgery and anesthesia, complication rates were comparable between the two groups, with only one wound infection reported in the open surgery group. However, the low incidence of complications limits a definitive comparison of safety outcomes.

Conclusion

Laparoscopic surgery for adnexal masses offers significant advantages over open surgery in terms of reduced blood loss, faster recovery, and lower postoperative morbidity. It represents a favorable surgical option for improving short-term perioperative outcomes; however, larger studies are required to establish definitive conclusions regarding comparative safety.

## Introduction

Adnexal masses, defined as any mass arising from the ovaries, fallopian tubes, or surrounding tissues, are among the most common gynecological problems affecting women of all age groups [1]. These lesions may be benign, malignant, or non-neoplastic and are often detected incidentally during clinical examination or imaging [1]. The incidence of adnexal masses in pregnancy is reported to be approximately two to 20 in 1000 pregnancies [2]. Because ovarian cancer accounts for a substantial proportion of cancer-related mortality among women, adnexal masses warrant careful evaluation and management [3]. Common presenting symptoms include abdominal or pelvic pain, abdominal distension, bloating, urinary frequency, and weight loss [1]. Persistence of such symptoms for more than two weeks or failure to respond to appropriate treatment necessitates further evaluation. Transvaginal ultrasonography is the standard initial imaging modality for assessment, supported by clinical examination and relevant laboratory investigations [4].

The majority of adnexal masses originate from the ovaries and fallopian tubes. Ovarian masses occur in nearly 6.9% of women, with ovarian neoplasms accounting for about 4.7% [5]. Fallopian tube involvement is commonly seen in pelvic inflammatory disease, whereas primary fallopian tube carcinoma is rare, with an incidence ranging from 0.1% to 1.8% [6]. While many adnexal masses are asymptomatic and benign and may be managed conservatively, those with suspicious features or associated symptoms often require surgical intervention [7]. The choice of surgical approach depends on factors such as the nature of the mass, patient characteristics, and available expertise.

With advances in minimally invasive surgery, laparoscopy has become a widely accepted approach for the management of adnexal masses [8]. Laparoscopic surgery offers several advantages including smaller incisions, reduced postoperative pain, lower risk of infection, shorter hospital stay, and faster recovery [9]. However, the procedure may be technically challenging in patients with dense adhesions, obesity, prior pelvic surgery, or endometriosis, and may be associated with longer operative time and higher instrument costs [10]. In contrast, laparotomy remains a commonly used technique, particularly for large or suspicious masses, due to its shorter operative time and lower equipment cost, though it is associated with greater blood loss, longer hospital stay, and increased postoperative morbidity [11-13].

Despite these findings, the optimal surgical approach for adnexal masses continues to be debated due to differences in patient profiles, disease characteristics, and healthcare settings. Therefore, the present study aimed to compare laparoscopic adnexal surgery and open adnexal surgery for adnexal masses. The primary outcome measure was postoperative complications (postoperative comorbidities), while secondary outcome measures included duration of surgery, duration of anesthesia, intraoperative blood loss, postoperative hemoglobin levels, analgesic requirement, need for blood transfusion, and duration of hospital stay.

This research work was originally conducted as part of a postgraduate dissertation submitted to the Department of Obstetrics and Gynecology, Shri B. M. Patil Medical College, Hospital & Research Centre, Bijapur Lingayat Development Education (BDLE) (Deemed to be University), Vijayapura, Karnataka, India.

## Materials and methods

This randomized controlled trial was conducted in the Department of Obstetrics and Gynecology at Shri B.M. Patil Medical College and Hospital, Vijayapura, Karnataka, India, from March 2024 to October 2025. Women attending the Obstetrics and Gynecology outpatient department (OPD) with a diagnosis of adnexal mass requiring surgical management were screened for eligibility. The objectives, procedures, potential benefits, and risks of the study were explained to all participants in their local language, and written informed consent was obtained prior to enrollment. Ethical approval was obtained from the Institutional Ethics Committee of Shri B. M. Patil Medical College and Hospital (IEC No: BLDE(DU)/IEC-SBMPMC/112/2023-24), and the trial was registered with the Clinical Trials Registry of India (CTRI No: CTRI/2025/03/082155). The study was conducted in accordance with the principles of the Declaration of Helsinki and Good Clinical Practice guidelines.

The sample size was calculated based on anticipated proportions of post-operative complications following laparoscopic and open surgery for adnexal masses, which were 7.4% and 37.5%, respectively, as reported by Pulcinelli et al. [14]. Using these values, a minimum of 30 patients per group (total sample size of 60) was required to achieve 80% power to detect a significant difference between the two groups at a two-sided alpha error of 0.05. Sample size estimation was performed using G*Power software (Ver 3.1.9.7, Heinrich-Heine-Universität Düsseldorf, Düsseldorf, Germany; z tests - proportions: difference between two independent proportions).

Women with adnexal masses who came to the OPD, confirmed by ultrasonography and scheduled to undergo either laparoscopic surgery or laparotomy, were included in the study. Patients were excluded if they had abnormal uterine bleeding, uterine fibroids, gynecological malignancies such as carcinoma cervix or carcinoma endometrium, hemodynamic instability, or major medical comorbidities, including cardiac failure and liver failure.

Eligible participants were randomly allocated into two groups using a computer-generated random number sequence with equal allocation ratio (1:1). Allocation concealment was ensured using sequentially numbered, opaque, sealed envelopes, which were opened only after patient enrollment. Due to the nature of the surgical interventions, blinding of the surgeon and participants was not feasible. However, outcome assessment and data analysis were performed by investigators who were not involved in the surgical procedure.

Group A consisted of patients who underwent laparoscopic surgery for adnexal masses, while Group B comprised patients who underwent laparotomy (open surgery). Laparoscopic procedures were performed under general or spinal anesthesia using standard port placement. Pneumoperitoneum was established by either the closed (Veress needle) [15] or open (Hasson) technique [16], following which a systematic inspection of the pelvis and abdomen was carried out. The adnexal mass was evaluated intraoperatively, and the appropriate surgical procedure, including cystectomy, salpingectomy, or salpingo-oophorectomy, was performed based on the nature and extent of the pathology. Laparotomy was performed under spinal or general anesthesia through a Pfannenstiel or midline abdominal incision, depending on the size of the mass and intraoperative findings. After entering the peritoneal cavity, the adnexal mass was identified and assessed, and the required surgical procedure like cystectomy, salpingectomy, or salpingo-oophorectomy was carried out. Hemostasis was achieved in all cases, and the abdomen was closed in layers.

All patients underwent standardized preoperative evaluation including detailed medical and gynecological history, general and pelvic examination, routine hematological and biochemical investigations, and pelvic ultrasonography to characterize the adnexal mass. Hemoglobin levels were recorded preoperatively and postoperatively. The type of anesthesia administered, duration of anesthesia, duration of surgery, and estimated intraoperative blood loss were documented for all cases. Blood loss was estimated based on suction bottle measurements after subtracting irrigation fluid and by standardized mop weight (weight of soaked mop minus dry mop). The primary outcome measure was post-operative comorbidities, while secondary outcome measures included intraoperative blood loss, duration of surgery, type and duration of anesthesia, postoperative hemoglobin levels, analgesic requirement, need for blood transfusion, and duration of hospital stay.

Postoperative complications were defined as any adverse clinical event occurring during hospitalization according to the Clavien-Dindo classification [17]. Postoperatively, all patients received a standardized postoperative care protocol and were monitored daily for pain using routine clinical assessment, requirement of analgesics, signs of infection, and other complications. Postoperative pain management was standardized across both groups. All patients received intravenous paracetamol (1 g every eight hours) for the first 24 hours, followed by oral analgesics as required. Additional analgesia (e.g., tramadol 50-100 mg intravenously) was administered based on patient-reported pain and clinical judgment. Analgesic requirement was recorded as the total number of doses administered until discharge. 

Patients were discharged based on predefined criteria, including hemodynamic stability, adequate pain control on oral analgesics, ability to ambulate independently, absence of fever or active complications, and tolerance of oral diet. The need for blood transfusion, length of hospital stay, and any adverse events such as wound infection were documented.

**Figure 1:**
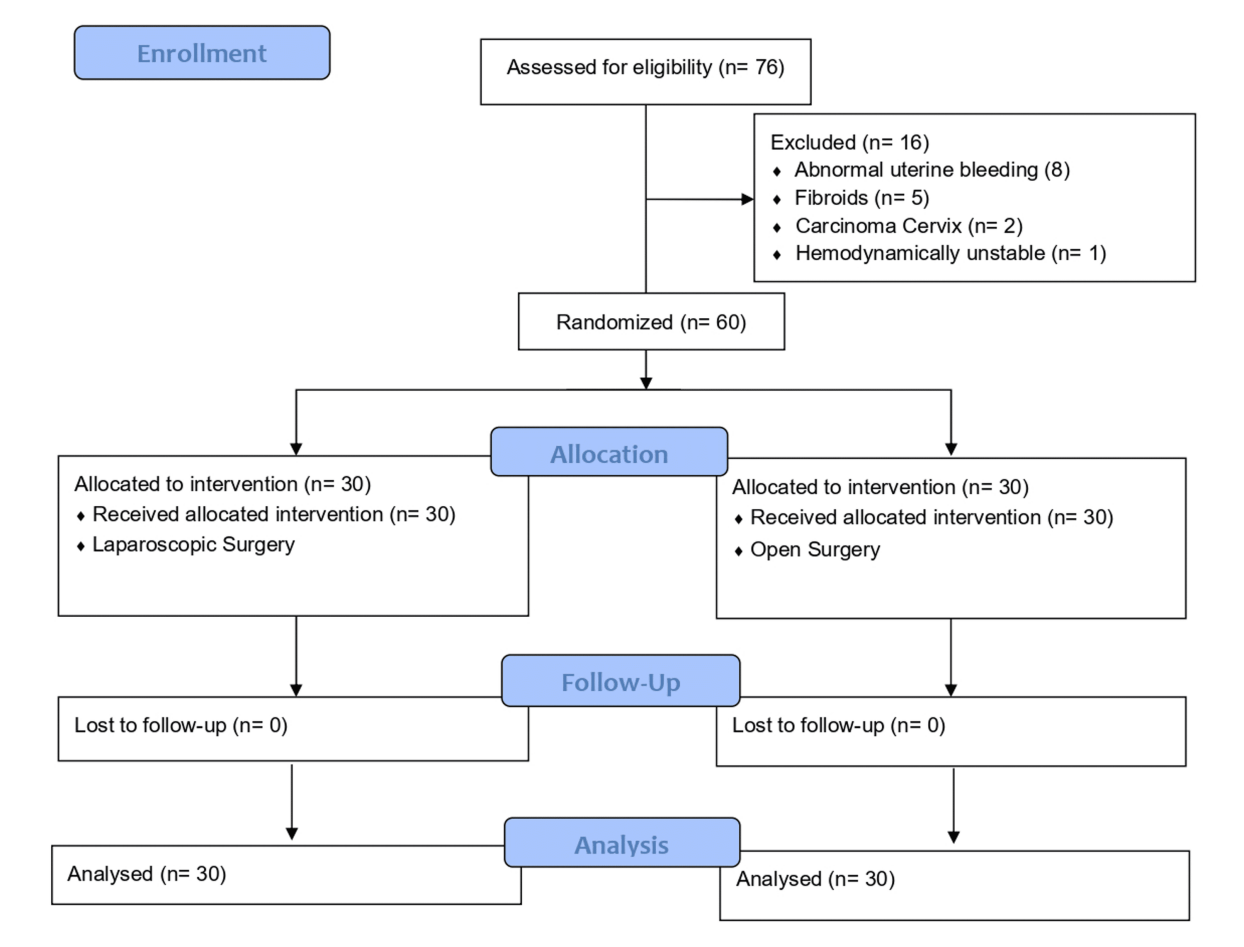
CONSORT Flow Diagram CONSORT: Consolidated Standards of Reporting Trials.

Data were entered into Microsoft Excel (Microsoft Corp., Redmond, WA, United States) and analyzed using IBM SPSS Statistics for Windows, Version 26 (Released 2019; IBM Corp., Armonk, New York, United States). Categorical variables were expressed as N (%) and continuous variables as mean ± standard deviation. Comparison between the two groups was done using the Chi-square test for categorical variables and suitable parametric tests for continuous variables. A p-value of less than 0.05 was considered statistically significant. All randomized participants were included in the final analysis, and results were reported following Consolidated Standards of Reporting Trials (CONSORT) guidelines [18] for randomized controlled trials (Figure 1).

## Results

A total of 76 patients were assessed for eligibility, of whom 16 were excluded due to predefined exclusion criteria. Sixty patients were randomized equally into two groups: laparoscopic surgery (n=30) and open surgery (n=30). The two groups were comparable with respect to baseline characteristics. Most patients in both groups were young adults, with the largest proportion in the 21-30 year age group. The mean age of patients in the laparoscopic group was 35.27 ± 12.32 years, while that in the open surgery group was 32.77 ± 9.65 years. Urban residence predominated in both laparoscopic and open surgery groups, and housewives formed the major occupational category in each group. The majority of participants belonged to the middle socioeconomic class, followed by the lower-middle class. No statistically significant differences were observed between the groups for age, residence, occupation, or socioeconomic status, indicating that the study groups were well matched at baseline (Table 1).

**Table 1:** Baseline demographic and clinical characteristics of study groups (n=60) Data are presented as frequency and percentage for categorical variables. Comparison between laparoscopic surgery and open surgery groups was performed using the Chi-square (χ²) test. n: Number of participants; χ²: Chi-square test. A p value of less than 0.05 was considered statistically significant.

Category	Variable	Laparoscopic surgery (n=30)	Open surgery (n=30)	χ² value	p value
Age group (years)	21–30	12 (40.0%)	12 (40.0%)	1.36	0.851
	31–40	7 (23.3%)	9 (30.0%)		
	41–50	4 (13.3%)	5 (16.7%)		
	51–60	6 (20.0%)	3 (10.0%)		
	61–70	1 (3.3%)	1 (3.3%)		
Residence	Urban	18 (60.0%)	16 (53.3%)	0.27	0.603
	Rural	12 (40.0%)	14 (46.7%)		
Occupation	Self-employed	6 (20.0%)	5 (16.7%)	0.60	0.742
	Employed	9 (30.0%)	11 (36.7%)		
	Housewife	15 (50.0%)	14 (46.7%)		
Socioeconomic status	Upper	4 (13.3%)	3 (10.0%)	1.49	0.688
	Middle	15 (50.0%)	16 (53.3%)		
	Lower-middle	8 (26.7%)	7 (23.3%)		
	Lower	3 (10.0%)	4 (13.3%)		

Prior surgical history showed a similar distribution in both groups, with previous lower segment caesarean section (LSCS) seen in a small proportion of cases and prior tubectomy noted only in the open surgery group. Ovarian cyst was the most common adnexal pathology in both groups, occurring more frequently in the laparoscopic group. Conditions such as ectopic pregnancy and ovarian torsion were relatively more common in the open surgery group, whereas benign adnexal masses were more often managed laparoscopically. With regard to the type of surgery performed, cystectomy was the most frequent procedure overall, particularly in the laparoscopic group, while more extensive procedures such as total abdominal hysterectomy with bilateral salpingo-oophorectomy were more commonly performed in the open surgery group. These differences were not statistically significant (Table 2).

**Table 2:** Surgical profile of study participants (n=60) Data are presented as frequency and percentage. Intergroup comparison between laparoscopic and open surgery groups was performed using the Chi-square (χ²) test. LSCS: Lower Segment Caesarean Section; TAH: Total Abdominal Hysterectomy; BSO: Bilateral Salpingo-oophorectomy; n: Number of participants; χ²: Chi-square test. A p value of less than 0.05 was considered statistically significant.

Category	Variable	Laparoscopic surgery (n=30)	Open surgery (n=30)	χ² value	p value
Prior surgical history	Previous LSCS	4 (13.3%)	3 (10.0%)	0.16	0.687
	Previous tubectomy	0 (0.0%)	2 (6.7%)	2.06	0.150
Type of adnexal mass	Ovarian cyst	18 (60.0%)	10 (33.3%)	7.20	0.303
	Ectopic pregnancy	1 (3.3%)	6 (20.0%)		
	Hydrosalpinx	3 (10.0%)	2 (6.7%)		
	Tubo-ovarian abscess	1 (3.3%)	2 (6.7%)		
	Mucinous cystadenoma	3 (10.0%)	4 (13.3%)		
	Serous cystadenoma	2 (6.7%)	2 (6.7%)		
	Ovarian torsion	2 (6.7%)	4 (13.3%)		
Type of surgery performed	Cystectomy	19 (63.3%)	10 (33.3%)	7.08	0.132
	Salpingectomy	4 (13.3%)	6 (20.0%)		
	Salpingo-oophorectomy	4 (13.3%)	5 (16.7%)		
	TAH + BSO	1 (3.3%)	6 (20.0%)		
	Oophorectomy	2 (6.7%)	3 (10.0%)		

Significant differences were observed in intra-operative parameters between the two groups. Spinal anesthesia was the predominant mode in both groups, but general anesthesia was used more often in laparoscopic cases. The duration of anesthesia and duration of surgery differed significantly, with laparoscopic procedures more frequently falling in the 91-120 minute range due to procedural complexity and adhesions, whereas a higher proportion of open surgeries were completed within 90 minutes. Blood loss was substantially lower in the laparoscopic group, where most patients had losses within 100-300 mL, while open surgery was associated with higher volumes of blood loss, with many patients exceeding 300 mL. These findings indicate a clear intra-operative advantage for laparoscopic surgery (Table 3).

**Table 3:** Intra-operative parameters of the study groups (n=60) Data are presented as frequency and percentage. Differences between laparoscopic and open surgery groups were analyzed using the Chi-square (χ²) test. min: Minutes; ml: Milliliters; n: Number of participants; χ²: Chi-square test. A p value of less than 0.05 was considered statistically significant.

Parameter	Variable	Laparoscopic surgery (n=30)	Open surgery (n=30)	χ² value	p value
Type of anesthesia	Spinal	25 (83.3%)	29 (96.7%)	4.32	0.038
	General	5 (16.7%)	1 (3.3%)		
Duration of anesthesia (min)	≤90	3 (10.0%)	12 (40.0%)	10.92	0.004
	91–120	17 (56.7%)	8 (26.7%)		
	121–150	10 (33.3%)	10 (33.3%)		
Duration of surgery (min)	≤90	14 (46.7%)	17 (56.7%)	14.82	<0.001
	91–120	16 (53.3%)	12 (40.0%)		
	121–150	0 (0.0%)	1 (3.3%)		
Blood loss (ml)	<100	1 (3.3%)	0 (0.0%)	42.76	<0.001
	101–200	19 (63.3%)	0 (0.0%)		
	201–300	10 (33.3%)	6 (20.0%)		
	301–400	0 (0.0%)	12 (40.0%)		
	401–500	0 (0.0%)	7 (23.3%)		
	>600	0 (0.0%)	5 (16.7%)		

Post-operative outcomes also favored the laparoscopic approach. Pre-operative hemoglobin levels were comparable between groups, but post-operative hemoglobin was significantly higher in the laparoscopic group. None of the laparoscopic patients required blood transfusion, whereas a proportion of open surgery patients did. Analgesic requirement was markedly lower after laparoscopic surgery, with most patients requiring fewer doses, while the majority of open surgery patients required five or more doses. Hospital stay was significantly shorter in the laparoscopic group, with all patients discharged within three days, compared to prolonged hospitalization in the open surgery group. Post-operative complications were minimal in both groups, with only an isolated case of wound infection in the open surgery group. Due to the very low number of complications observed, meaningful statistical comparison of safety outcomes is limited (Table 4).

**Table 4:** Post-operative outcomes among study groups (n=60) Categorical variables are presented as frequency and percentage, while continuous variables are presented as mean ± standard deviation (SD). Categorical variables were compared using the Chi-square (χ²) test, and continuous variables were compared using the independent Student’s t-test. A p value of less than 0.05 was considered statistically significant. SD: Standard Deviation; g/dL: Grams per deciliter; n: Number of participants; χ²: Chi-square test; t: Student’s t-test.

Outcome	Variable	Laparoscopic surgery (n=30)	Open surgery (n=30)	Statistical value	p value
Hemoglobin (g/dL) (Mean ± SD)	Pre-operative	11.09 ± 0.77	11.15 ± 1.46	t = 0.20	0.84
	Post-operative	10.54 ± 0.82	9.75 ± 1.43	t = 2.62	0.021
Blood transfusion	Yes	0 (0.0%)	5 (16.7%)	χ² = 5.61	0.018
Analgesic requirement (doses)	2–3	16 (53.3%)	0 (0.0%)	χ² = 46.21	<0.001
	4	14 (46.7%)	1 (3.3%)		
	≥5	0 (0.0%)	29 (96.7%)		
Hospital stay (days)	2–3	30 (100%)	0 (0.0%)	χ² = 60.00	<0.001
	4–5	0 (0.0%)	25 (83.3%)		
	≥6	0 (0.0%)	5 (16.7%)		
Post-operative complication	Wound infection	0 (0.0%)	1 (3.3%)	χ² = 1.02	0.313

## Discussion

The demographic profile of women with adnexal masses observed in this study is consistent with that reported in previous literature, with a clear predominance in the reproductive age group. Nouri et al. reported a mean age of 36 years and found no statistically significant difference in age between the laparoscopy and laparotomy groups, which is comparable to the age distribution seen in the present study [13]. Similarly, Dutta et al. reported a mean age of 37.2 years, with most patients belonging to the third and fourth decades of life, emphasizing that benign adnexal pathology predominantly affects women of reproductive age [19]. They also observed that a substantial proportion of adnexal masses are detected incidentally, underscoring the importance of fertility preservation and minimally invasive surgical strategies in this patient population.

Regarding operative duration, Nouri et al. reported a longer mean operative time for laparoscopy (141 ± 41 minutes) compared with laparotomy (120 ± 36 minutes) [13]. In contrast, Dutta et al. found shorter operative times for laparoscopic adnexal surgery, suggesting that operative duration is influenced by surgeon experience and institutional protocols [19]. Despite variability in operative time, most studies consistently demonstrate lower blood loss with laparoscopy. Nouri et al. found significantly reduced blood loss in laparoscopic procedures (p<0.001), and Dutta et al. reported a mean blood loss of 44.9 mL with laparoscopic techniques [13,19]. The findings of the present study are consistent with these observations, demonstrating significantly reduced intraoperative blood loss and improved perioperative outcomes in the laparoscopic group.

Anesthesia-related parameters and postoperative pain outcomes reported in the literature also favor laparoscopy. Nouri et al. observed longer anesthesia duration for laparoscopic surgery (179 ± 42 minutes) compared with laparotomy (150 ± 36 minutes) [13]. Dutta et al. similarly described improved postoperative comfort and earlier ambulation in laparoscopic patients [19]. These findings indicate that although laparoscopy may require slightly longer anesthesia time, it provides significant advantages in terms of postoperative recovery. In the present study, postoperative recovery was assessed using analgesic requirement, which was significantly lower in the laparoscopic group, further supporting improved postoperative recovery.

Hospital stay and complication profiles further distinguish the two approaches in favor of laparoscopy. Nouri et al. reported a significantly shorter hospital stay in laparoscopic patients (29 ± 9 hours) compared with laparotomy patients (44 ± 7 hours) [13]. Dutta et al. and Talwar et al. also documented shorter hospitalization following laparoscopic surgery [19,20]. Pulcinelli et al. reported higher postoperative complication rates in laparotomy (37%) compared with laparoscopy (7%), along with longer hospital stay (11 days vs. five days) [14]. Nouri et al. observed lower transfusion requirements in laparoscopic cases, further reflecting reduced intraoperative blood loss and improved perioperative safety [13]. In our study, while postoperative complications were minimal in both groups, the very low event rate limits meaningful comparison of safety outcomes between the two approaches.

Strengths and limitations

The strengths of the present study include its randomized controlled design, which minimizes selection bias and enhances the internal validity of comparisons between laparoscopic surgery and laparotomy in the management of adnexal masses. In addition, a comprehensive range of intraoperative and postoperative outcomes was evaluated, including duration of surgery, blood loss, duration of anesthesia, analgesic requirement, hospital stay, hemoglobin changes, blood transfusion requirement, and postoperative complications, allowing a holistic assessment of surgical safety and efficacy.

However, certain limitations must be acknowledged. The relatively small sample size (n=60) may limit statistical power and the ability to detect infrequent complications or subtle differences between the two surgical approaches. Importantly, the study was underpowered to detect differences in rare postoperative complications due to the very low number of events observed, and therefore conclusions regarding comparative safety should be interpreted with caution. As the study was conducted at a single center, the findings may not be fully generalizable to other settings with different patient populations or levels of surgical expertise. The analysis was restricted to short-term postoperative outcomes, and long-term parameters such as recurrence, adhesion formation, chronic pain, fertility outcomes, and quality of life were not assessed. Additionally, certain outcomes, such as analgesic requirement and duration of hospital stay, although standardized, may still be influenced by clinician judgment and institutional practices. Furthermore, although operative duration and hospital stay were recorded, a formal cost-effectiveness analysis was not performed. Nevertheless, as most participants were of reproductive age, the results are clinically relevant to routine gynecological practice, particularly in guiding fertility preservation and minimally invasive surgical strategies.

## Conclusions

Laparoscopic surgery for adnexal masses offers clear advantages over open surgery in terms of reduced intraoperative blood loss, lower postoperative analgesic requirement, shorter hospital stay, better preservation of postoperative hemoglobin levels, decreased need for blood transfusion, and improved short-term perioperative recovery outcomes. Although laparoscopic procedures may require slightly longer operative and anesthesia times, these disadvantages are outweighed by the benefits of faster recovery and improved perioperative outcomes. Laparoscopy may be considered a favorable surgical option in appropriately selected patients; however, larger studies are required to draw definitive conclusions regarding comparative safety.
